# Control of Biofilms with the Fatty Acid Signaling Molecule *cis*-2-Decenoic Acid

**DOI:** 10.3390/ph8040816

**Published:** 2015-11-25

**Authors:** Cláudia N. H. Marques, David G. Davies, Karin Sauer

**Affiliations:** 1 Department of Biological Sciences, Binghamton University, Binghamton, NY 13902, USA; E-Mails: dgdavies@binghamton.edu (D.G.D.); ksauer@binghamton.edu (K.S.); 2 Binghamton Biofilm Research Center (BBRC), Binghamton University, Binghamton, NY 13902, USA

**Keywords:** biofilms, biofilm dispersion, persister cells, signaling molecules, bacterial physiology, *cis*-2-decenoic acid

## Abstract

Biofilms are complex communities of microorganisms in organized structures attached to surfaces. Importantly, biofilms are a major cause of bacterial infections in humans, and remain one of the most significant challenges to modern medical practice. Unfortunately, conventional therapies have shown to be inadequate in the treatment of most chronic biofilm infections based on the extraordinary innate tolerance of biofilms to antibiotics. Antagonists of quorum sensing signaling molecules have been used as means to control biofilms. QS and other cell-cell communication molecules are able to revert biofilm tolerance, prevent biofilm formation and disrupt fully developed biofilms, albeit with restricted effectiveness. Recently however, it has been demonstrated that *Pseudomonas aeruginosa* produces a small messenger molecule *cis*-2-decenoic acid (*cis*-DA) that shows significant promise as an effective adjunctive to antimicrobial treatment of biofilms. This molecule is responsible for induction of the native biofilm dispersion response in a range of Gram-negative and Gram-positive bacteria and in yeast, and has been shown to reverse persistence, increase microbial metabolic activity and significantly enhance the cidal effects of conventional antimicrobial agents. In this manuscript, the use of *cis*-2-decenoic acid as a novel agent for biofilm control is discussed. Stimulating the biofilm dispersion response as a novel antimicrobial strategy holds significant promise for enhanced treatment of infections and in the prevention of biofilm formation.

## 1. Introduction

Biofilms are dynamic communities of closely associated microbial cells embedded within a hydrated extracellular polymeric substance (EPS) on an air-liquid interface, or adherent to inert (abiotic) or living surfaces and constitute the major proportion of bacterial biomass in nature [1]. The EPS of biofilm communities is composed of any combination of polysaccharides, nucleic acids, proteins, lipids, and under certain conditions, humic substances [2,3,4,5,6]. Biofilms are formed in a sequential manner [7,8,9,10], initiated by the reversible attachment of free-floating cells to a surface [11]. A transition to irreversible attachment is mediated by a variety of direct interactions with the substratum and is generally associated with the onset of the production of EPS which entraps bacteria and results in cell-cell aggregation and the formation of microcolonies [12,13,14,15,16]. As microcolonies grow, their resident bacteria alter their physiological state toward a biofilm-specific phenotype, described as maturation stages I and II [7,8,9,17,18]. When biofilm microcolonies begin to experience limited transport with the bulk liquid, the bacteria within the microcolony may undergo a final switch in phenotype from a sessile to a mobile state, releasing cells through breaches formed in the microcolony wall in a process called dispersion [7,19].

As three dimensionally structured communities, biofilms encompass a variety of environmental microniches characterized by bacteria with differences in metabolic activity, growth rate, nutrient utilization, and the expression of surface molecules and virulence factors [20,21,22,23,24,25,26,27,28,29]. The behavior of bacteria within a biofilm has been shown in many cases to be governed by secreted chemical signals that are used in cell-to-cell communication. An example of such behavioral modification is demonstrated by *Pseudomonas aeruginosa*, which as a wild-type forms highly structured heterogeneous biofilms, but in strains lacking the ability to produce the quorum sensing (QS) inducer 3-oxododecanoyl-HSL, forms biofilms displaying a flat, densely packed and homogeneous architecture [17]. Likewise, a *P. aeruginosa* *pqs* mutant, deficient in the production of 2-heptyl-3-hydroxy-4-quinolone (*Pseudomonas* quinolone signal—PQS) has been observed to form thin biofilms containing reduced levels of extracellular DNA, compared to the wild-type strain [6,30,31,32]. In addition to influencing biofilm architecture, bacterial cell-to-cell signaling within a biofilm can provide cues about local environmental conditions, modulate inter-species and intra-species interactions with other individuals within the community, and allow coordinated responses to enable group-level expression of virulence and bacterial persistence. Cell-cell signaling in bacteria is accomplished through the production and detection of small diffusible signaling molecules of different chemical classes, that can be produced by Gram-negative bacteria, Gram-positive bacteria, or both Gram-positive and Gram-negative. To date, more than 50 such molecules have been identified and include: autoinducer-1 (AI-1) also known as *N*-acylhomoserine lactones (*N*-AHL), autoinducer-2 (AI-2) a furanosyl borate, PQS, oligopeptides (5–10 amino acid cyclic thiolactone) known as autoinducer peptides (AIP), and short-chain fatty acids which are typically unsaturated at the number 2 carbon in a *cis* configuration [33,34,35,36,37]. Secondary messengers provide a further level of regulation and include c-di-GMP, known to regulate biofilm development, with high intercellular levels of this molecule inducing the production of adhesins and EPS, and low levels having the opposite effect [38,39,40]. In *P. aeruginosa*, biofilm formation has been linked to elevated c-di-GMP levels, while dispersion is coincident with significantly reduced c-di-GMP [38,41].

One of the hallmark characteristics of biofilms is their profound tolerance to antimicrobial agents [42,43,44], which results in persistent infections and renders biofilms difficult to control [42,45]. Biofilms are typically 10 to 1000 times less susceptible to antimicrobials when compared to their planktonic counterparts [22,46,47,48]. This resistance to killing, referred to here as “biofilm tolerance” is distinct from specific resistance conferred by mutation of an antimicrobial target or the acquisition of a plasmid-borne resistance marker [46,49,50,51,52]. Instead, biofilm tolerance is observed as a generalized reduction in susceptibility to killing by essentially any antimicrobial agent. This tolerance can be overcome, in part, by significantly increasing the concentration of antimicrobial, suggesting that specific resistance is not a component of biofilm tolerance, and that cells derived from biofilms do not exhibit specific tolerance. Furthermore, biofilm tolerance may be overcome by inducing biofilm bacteria to mount a dispersion response, resulting in the evacuation of bacterial cells from the biofilm; a behavior that re-establishes susceptibility of the participating bacteria to antimicrobial agents [53,54,55].

Biofilm tolerance arises from a combination of factors that are associated with the unique environment bacteria experience within the confines of a biofilm, including: failure of the antimicrobial to penetrate throughout the biofilm matrix [21,56,57,58], reduced growth rates by the resident bacteria, a wide range of non-specific protective adaptations (such as enhanced efflux) associated with the biofilm phenotype [59,60,61,62,63], and the formation of a persister cell sub-population [64,65,66,67]. Biofilm tolerance further extends to resistance to killing by the immune system, with bacteria deriving protection against immune cells by being embedded within the mesh-like network of EPS [68].

Considering the multifactorial nature of biofilm resistance, it is apparent that strategies to eradicate biofilms have to address all the above mechanisms simultaneously [21]. While there are numerous ongoing efforts to address the biofilm problem, the most promising strategies include those aimed at manipulating the mode of growth, by either preventing biofilms from forming, or by disrupting existing biofilms. This is further supported by the finding that dispersion, the evacuation of bacterial cells from the biofilm, coincides with the reinstatement of antibiotic sensitivity [53,54,55]; suggesting that the potential to overcome the biofilm resistance mechanisms or to induce the transition of biofilm bacteria from a resistant to a susceptible phenotype would likely result in enhanced treatment options in fighting biofilm infections.

In this review, we will discuss a novel strategy to enhance the susceptibility of biofilm cells by subverting the normal biological processes that maintain biofilm integrity and promote biofilm tolerance. This strategy takes advantage of the regulatory networks that are controlled by inter and intra-species signaling and will focus primarily upon the cross-kingdom signaling molecule *cis*-2-decenoic acid, a fatty acid signal that has been shown to prevent biofilm development, induce biofilm dispersion, cause global changes in cellular phenotype, modulate bacterial virulence and override microbial persistence.

## 2. Jamming Bacterial Communication

Intercellular signaling regulates functions contributing to persistence and virulence of many bacterial pathogens. Thus, signaling interference holds significant promise as the basis of novel therapeutic strategies to improve the outcome of bacterial, and in particular biofilm infections [69,70,71]. QS plays a vital role in the regulation of virulence and biofilm-related behaviors both in the natural environment and in persistent infections [72,73], and is currently considered to be the main target as means of control of biofilm infections in the lung infections of cystic fibrosis patients, as well as other infections. Disruption of QS can be achieved by reducing the *N-*acylhomoserine lactone (*N*-AHL) synthase activity, inhibiting the production of the QS molecules, degrading *N*-AHLs (the most used), and using synthetic compounds that mimic QS inducer molecules [33]. Below, we provide several examples of quorum quenching, where interference with QS signalling occurs, leading to the arrest of biofilm development, disaggregation of established biofilms, and reduction of virulence.

*P. aeruginosa*, is an opportunistic microorganism found in a variety of niches and able to cause acute and chronic infections such as those found in the lungs of cystic fibrosis patients [74,75,76,77]. It is thus, a principal model microorganism used in the study of biofilms and biofilm cell behavior, has one of the most extensively investigated QS systems, and has been found to have approximately 10% of its genome dedicated to signalling [78]. *P. aeruginosa* contains one of the most complex QS systems known in bacteria, where an interconnected signaling cascade coordinates virulence, persistence, the transition to sessile growth, and biofilm development [79]. Three separate and inter-related QS systems are used by *P. aeruginosa*. These systems are regulated in a hierarchical manner with LasI/LasR positively regulating the RhlI/RhlR system, while the PQS system is regulated by both the LasI/LasR and RhlI/RhlR systems [30,73,80]. Considering that establishment of infections by *P. aeruginosa* is dependent on the LasI/LasR system, with LasR being required for QS signal binding and activation of the regulatory cascade, it is not surprising that the identification of LasR inhibitors has become a major focus in *P. aeruginosa* research [34]. The LasR inhibitors comprise N-AHL signaling molecules that have been chemically altered with respect to the acyl side chain, and the lactone ring [34]. In addition to inhibiting LasR, some of these molecules have been found to be potent inhibitors of RhlR [81,82]. For instance, *meta*-bromothiolactone was shown to inhibit pyocyanin production (a virulence factor) and biofilm formation, and to protect *Caenorhabditis elegans* and human lung epithelial cells from killing by *P. aeruginosa* [83]. Additional N-AHL inhibitors comprise various naturally occurring products such as furanones and patulin and their derivatives [83,84,85,86,87,88], with synthetic furanones having been demonstrated to significantly attenuate *P. aeruginosa* lung infections [83].

Modulators of the secondary messenger molecule c-di-GMP have also previously been described. The molecules LP 3134, LP 3145, LP 4010 and LP 1062 were found to inhibit the diguanylate cyclases (DCG), WspR from *P. aeruginosa* and tDGC from *Thermotoga maritime*; both required for c-di-GMP synthesis [89]. The inactivation of these DGCs resulted in biofilm inhibition of *P. aeruginosa* and *Acinetobacter baumannii* [89]. In addition, the compounds ebselen and ebselen oxide were also found to inhibit c-di-GMP by covalently modifying cysteine residues and inhibiting the binding of c-di-GMP to particular receptors, ultimately regulating biofilm development [90].

Most of these QS inhibitors are naturally occurring and show promise as therapeutic agents due to their cross species activity. For example, the QS modulators curcumin and its derivatives have been shown to reduce biofilm growth of *P. aeruginosa*, *Escherichia coli* and *Vibrio harveyi*, while also reducing pathogenicity and the accumulation of microorganisms on wet surfaces (biofouling) [85]. A combination of quorum quenching metabolites (inhibitors of QS) with antimicrobials has been demonstrated to enhance the susceptibility of *P. aeruginosa* and other microorganisms [86,91]. In *Staphylococcus aureus*, a Gram-positive organism, an RNAIII-inhibiting peptide suppressed the staphylococcal TRAP/agr two-component regulatory systems [involved in biofilm formation and virulence), reduced biofilm formation *in vivo*, and prevented infections by methicillin-resistant *S. aureus* (MRSA) in rats [92]. Several anti-QS peptides have also led to the inhibition of biofilm formation in Gram-positive oral bacteria [93].

These examples underscore the importance of QS quenching in the control of biofilm infections. However, molecules involved in QS have different chemical compositions, different targets, and vary significantly from species to species, and to date, no universal molecule that is able to quench (or inhibit) all different QS systems has been found. While QS quenching shows promise as a strategy for combatting biofilms [94], this approach is limited due to the complex interactions between the signals of the various species, and the various mechanisms that control QS sensing [95].

## 3. Fatty Acid Signaling Systems

Fatty acid signals comprise a growing group of recently identified structurally related inducer molecules, which regulate a wide range of cellular functions. These signals have been identified in a range of Gram-positive and Gram-negative bacteria, as well as in the yeast *Candida albicans* (Table 1), and are now recognized as a unique chemical class of QS signals [96]. To readily distinguish these molecules from one another, a new nomenclature has been proposed where the methyl (Me) substitution, if present, and its position are indicated first, the number of carbons in the fatty acyl side-chain is indicated second, and the position of the double bond (Δ) is indicated last, for example: *cis*-11-methyl-2-dodecenoic acid is named 11-Me-C_12_:Δ^2^, where the methyl group is on carbon 11, in a 12 carbon fatty acid, with a double bond in the *cis* configuration between the number 2 and number 3 carbons (Table 1) [97].

Akin to *N*-AHLs and other cell-cell signaling molecules, fatty acid signals are involved in intra-species, inter-species, and cross-kingdom communication where they regulate bacterial growth, virulence, motility, polymer production, biofilm development, biofilm dispersion and persistence. The fatty acid signal 11-Me-C_12_:Δ^2^ in *Xanthomonas campestris* was the first member of this sub-class of *cis*-2-unsaturated fatty acids to be characterized and was named DSF for diffusible signal factor [98,99]. Interspecies signaling using fatty acids has been shown to occur between members of the *Burkholderia cepacia* complex [97], and between the members of *Xanthomonads* [96]. The *Xyllela fastidiosa* signal C14:Δ^2^, and the *Burkholderia cenocepacia* signals C_12_:Δ^2^ and 11-Me-C_12_:Δ^2,5^ are functional homologues of 11-Me-C_12_:Δ^2^ and both regulate biofilm formation and production of virulence factors [97,100]. Additionally, both signaling molecules contribute to the regulation of virulence factors, in response to elevated concentrations of 11-Me-C_12_:Δ^2^ produced by *Xanthomonas campestris*, *Stenotrophomonas maltophilia* and *Xylella* sp., leading to the disaggregation of flocs (clumps) present in planktonic cultures, acriflavin resistance and detoxification [101,102,103].

**Table 1:** Fatty acid signaling molecules with known functions in the various microorganisms.

Compound	New Nomenclature	Structure	Bacterial Species	Function	Reference
*cis*-11-Methyl-2-dodecenoic acid (DSF)	11-Me-C_12_:Δ^2^	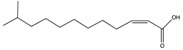	*Xanthomonas campestris*, *Xanthomonas oryzae*, *Stenotrophomonas maltophilia*, *Burkholderia multivorans*	Virulence, biofilm formation, floc disaggregation, microcolony formation, tolerance to antibiotics, detoxification, hyphal growth inhibition	[96,97,98,99,104,105]
*cis*-2-Dodecenoic acid (BDSF)	C_12_:Δ^2^	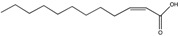	*Burkholderia cenocepacia*, *Burkholderia lata Burkholderia stabilis Burkholderia vietnamiensis Burkholderia dolorosa Burkholderia ambifaria Burkholderia anthina Burkholderia pyrrocinia B. multivorans*, *X. oryzae*	Virulence, hyphal growth inhibition	[97,104,106,107]
*cis*-2-Decenoic acid (*cis*-DA)	C_10_:Δ^2^	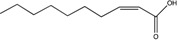	*Pseudomonas aeruginosa*	Biofilm formation, biofilm dispersion, persister cell formation, persister cell awakening, tolerance to antimicrobials.	[19,65,108,109,110]
*cis*-2-Tetradecenoic acid	C_14_:Δ^2^	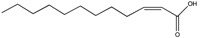	*Xylella fastidiosa*	Virulence and aggregation	[100]
*trans*-2-Decenoic acid (SDSF)	C_10_:Δ^2t^	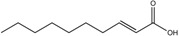	*Streptococcus mutans*	Hyphal growth inhibition	[111]
*cis*-11-Methyldodeca-2,5-dienoic acid (CDSF)	11-Me-C_12_:Δ^2,5^	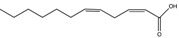	*B. multivorans*, *B. stabilis B. anthina*, *B. pyrrocinia*, *X. oryzae*	Hyphal growth inhibition	[97,104]
10-Methyldodecanoic acid	10-Me-C_12_	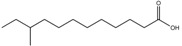	*S. maltophilia*	Stress tolerance and antibiotic tolerance	[105]
11-Methyldodecanoic acid	11-Me-C_12_	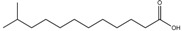	*S. maltophilia*	Stress tolerance and antibiotic tolerance	[105]
12-Methyltetradecanoic acid	12-Me-C_14_	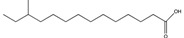	*Xylella fastidiosa*	Virulence, biofilm formation, motility	[112,113]
3-Hydroxypalmitic acid	3OH-PAME	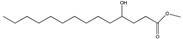	*Ralstonia solanacearum*	Virulence	[114]
Farnesoic acid	3,7,11-Me-C_12_:Δ^2t,6t,1^°^t^	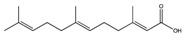	*Candida albicans*	Inhibition of germ tube formation	[115]

In *S. maltophilia*, 11-Me-C_12_:Δ^2^ production has been linked to changes in the lipopolysaccharide composition of LPS, increased protease synthesis, enhanced cell aggregation, biofilm microcolony formation and increased tolerance to antibiotics and heavy metals [116]. However, fatty acid signals do not only increase aggregative behavior or biofilm formation capability. For instance, 12-Me-C_14_ which was originally isolated from *Xylella fastidiosa*, has been found to abolish swarming motility and to reduce biofilm formation by *X. fastidiosa* and in *P. aeruginosa* [117]. The various fatty acids produced by *S. maltophilia* (Table 1) have also been demonstrated to have interspecies activity, and have been found to increase the stress tolerance and resistance of *P. aeruginosa* to antimicrobial peptides [118]. Production of fatty acid signals is not limited to bacteria, but is also found within the fungi where *C. albicans* has been shown to produce farnesoic acid which regulates the morphological transition from a yeast-form to a hyphal-form by inhibiting germ tube formation [96,115]. In addition to responding to its own fatty acid signal, *C. albicans* has been shown to respond to a variety of signals from other organisms, including C_12_:Δ^2^ from *B. cenocepacia*, C_10_:Δ^2t^ from *S. mutans*, 11-Me-C_12_:Δ^2^ from *X. campestris* and 11-Me-C_12_:Δ^2,5^ from *Burkholderia multivorans*, demonstrating the cross-kingdom capabilities of fatty acid signals [96,97,106,111].

## 4. The Fatty Acid System in *P. aeruginosa* and Its Role in Biofilm Control

The fatty acid signal *cis*-2-decenoic acid (C_10_:Δ^2^), which will be referred to subsequently as *cis*-DA, was recently identified in *P. aeruginosa* and found to act as a biofilm dispersion autoinducer [19]. The *cis*-DA signaling system has been shown to regulate 666 genes encoding proteins involved in motility, chemotaxis, cell attachment, TCA cycle, EPS and LPS synthesis and secretion, virulence, iron uptake, and respiration [119]. Current findings have shown the *dspI* gene (PA14_54640), a *PA0745* ortholog, to be required for the production of *cis*-DA [120]. Mutation of *dspI* in *P. aeruginosa* leads to a reduction of pyoverdine (a virulence factor) production, and to defective swarming motility [121]. However, the complete signaling transduction mechanism of the *cis*-DA system has yet to be fully elucidated. Most importantly and unlike other fatty acids and other signaling systems, *cis*-DA has been demonstrated to control biofilms by three different mechanisms: by impairing biofilm formation, by inducing biofilm dispersion [19], and by modulating the persister cell sub-population [65]. Additionally, *cis*-DA shows interspecies activity and also induces a dispersion response similar to that observed by *P. aeruginosa* in biofilms formed by *C. albicans* and various Gram-negative and Gram-positive bacteria, including other *Pseudomonads*, *E. coli*, *Streptococcus pyogenes*, *S. aureus*, *Klebsiella pneumoniae*, *Proteus mirabilis* and *Bacillus subtilis* [19]. Furthermore, *cis*-DA has been shown to induce biofilm dispersion in *Propionibacterium acnes* [122], *Actimomyces naeslundii*, *Lactobacillus casei* and *Streptococcus mutans* [123], when grown as single or mixed species biofilms*.* To date no other fatty acid has been shown to induce biofilm dispersion. However, 11-Me-C_12_:Δ^2^ has been demonstrated to disaggregate bacterial flocs of *X. campestris* [101].

### 4.1. Dispersion Induced by cis-2-Decenoic Acid as Means of Biofilm Control

Dispersion is one of the only bacterial behaviors known to be unique to biofilms, and is modulated by cell-to-cell communication. Biofilm dispersion is characterized by the release of bacteria from established biofilms, presumably to escape overcrowding or degraded environmental conditions. Autoinduction of dispersion occurs when microcolonies within a biofilm have attained a critical maximum size, and provides a mechanism by which bacteria can evacuate a microcolony, enter the bulk liquid environment, and disseminate to new sites for attachment [19]. The biofilm-specific nature of the dispersion response implies that drug development focusing on this behavior, could lead to an effective treatment of biofilm infections. The principal advantage to using drugs that target biofilm dispersion is that as bacteria (and fungi) transition from a biofilm to planktonic phenotype, the biofilm tolerance is reversed, enhancing susceptibility to antimicrobial agents and eliminating problems associated with antimicrobial penetration, the biofilm-specific phenotype and reduced growth rates [19,124]. Hence, exposure of biofilms to a dispersion inducer before and/or in combination with conventional antimicrobials would provide a novel mechanism for enhancing the activity of the treatments, through the disruption of existing biofilms [19,124]. Recent studies have shown that when compared to treatment with antimicrobials alone, targeting mature biofilms with antimicrobials in combination with *cis*-DA significantly reduces and removes the overall biofilm biomass in bacteria that are associated with biofilm infections and contamination of foods [108,109,110,125]. Exposure of pre-formed biofilms to different antimicrobials in the presence of *cis*-DA has been shown to lead to a reduction of the viability of MRSA *S. aureus* biofilm cells [109], and to significantly remove the biofilm biomass of *B. subtilis*, *Salmonella enterica*, *S. aureus* and *E. coli* from stainless steel and polystyrene surfaces, where they are known to cause food contamination [108]. In addition, co-exposure of *P. aeruginosa* PAO1 biofilm cells to antibiotics with *cis*-DA enhanced the killing efficacy of tobramycin or ciprofloxacin by greater than 1.5 Log, compared to treatment with the antibiotics alone [125]. It is notable that exposure to *cis*-DA alone shows no reduction in viability of bacteria [126], and has no cytotoxic effects on fibroblasts [109].

### 4.2. Persister Cell Control by Exposure to cis-DA

Persister cells are a subgroup (typically 0.0001%–1%) of a bacterial clonal population, consisting of dormant/semi-dormant specialised survivor cells that fail to succumb to treatment with antimicrobial agents, when the majority of the population shows susceptibility [64,65,66,127,128]. Various mechanisms have been proposed to explain persister cell formation, including down-regulation of genes involved in energy generation and cell maintenance [129,130], activation of the stringent response [131], and increased expression of toxin-antitoxin (TA) modules [64,132,133]. The growth rate distribution of cells within the differing microniches of a biofilm likely accounts for persister cell survival, with dormant or semi-dormant sub-populations existing at locations within the biofilm that are nutrient-limited, inducing a bacterial stress response [83,84].

The control of persister cells could be accomplished if this population were to be resuscitated to a metabolically active state, rendering them susceptible to challenge with antimicrobials. Exposure to metabolic stimuli such as—mannitol, glucose, fructose and pyruvate—have been shown to increase bacterial growth of *S. aureus* persister cells by more than 600 fold [134]. In addition to experiencing increased growth, exposure of *S. aureus* and *E. coli* persister cells to mannitol, glucose, fructose and pyruvate, also increased central metabolic activity as measured by increased respiration rates and membrane permeability [134]. This finding suggests that metabolic stimuli have the potential to resuscitate persister cells [134]. This is further supported by the finding that exposure to metabolic stimuli coincided with the eradication of *E. coli* persister cells by gentamicin [135]. In addition to metabolic stimuli, resuscitation of persister cells has been accomplished using QS inhibitors. The QS inhibitor (*Z*)-4-bromo-5-(bromomethylene)-3-methylfuran-2(5*H*)-one (BF8), known to inhibit AI-1 mediated QS through the inhibition of *lasB* expression, was shown to sensitize *P. aeruginosa* persister cells to ciprofloxacin and tobramycin, however the efficacy was reduced compared to exposure to metabolic stimuli has it did not result in complete cell eradication [136]. Although not used as a carbon source [65], *cis*-DA was found to significantly increase the number of cells recovered in agar plates [126], suggesting *cis*-DA to somehow trigger a transition from a dormant to an awake, likely more metabolically active susceptible state. This is supported by the finding that *P. aeruginosa* and *E. coli* persister cells obtained from planktonic and biofilm populations experience an additional decrease in viability (greater than 2 Log) to the point of eradication upon exposure to antimicrobials in combination with *cis*-DA compared to treatment with antimicrobials alone [65]. The effect of *cis-*DA is not limited to persister cells formed by Gram-negative organisms, as exposure of persister cells by the Gram-positive *S. aureus* to antibiotics in the presence of *cis*-DA also led to a significant decrease in cell viability, while antibiotics alone had no effect on the persister population [137]. In addition to increasing the metabolic status of persister cells and reverting their tolerant state, *cis*-DA also prevents persister cell formation. This is supported by the finding that the presence of *cis*-DA during persister cell isolation resulted in a 2-Log decrease in persister cell numbers in both *P. aeruginosa* and *E. coli* [65]. Thus, under the conditions tested challenge with *cis*-DA leads to a change in the cell’s metabolic status, resulting in a reduction of persister cell formation and a reversion of the persister cell’s tolerant state.

### 4.3. Prevention of Biofilm Formation by Exposure to cis-DA

**Figure 1:**
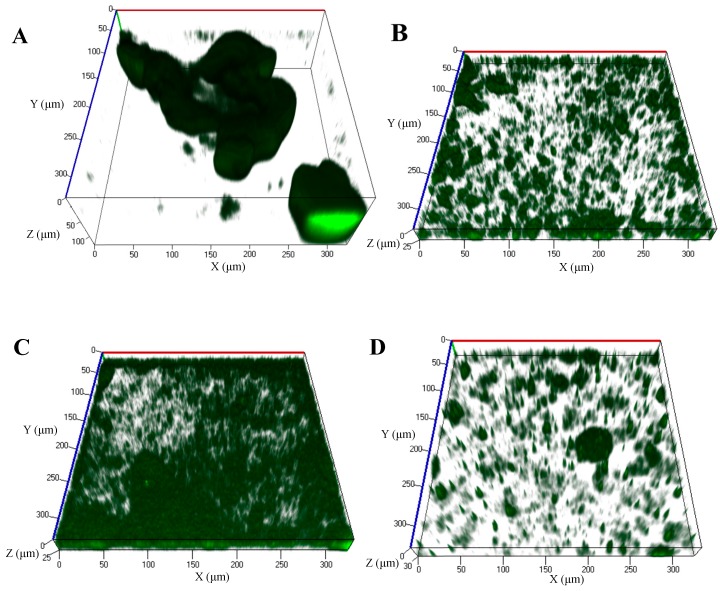
Biofilms of *P. aeruginosa* PA01 harboring the green fluorescence protein (GFP) expressing vector pMRP9-1 were grown in flow cell reactors in EPRI media, in the continuous presence of different concentrations of *cis*-2-decenoic acid (*cis*-DA). (**A**) Control (media); (**B**) 1 nM of *cis*-DA; (**C**) 1 μM of *cis*-DA; (**D**) 1 mM *cis*-DA. Following 96 h of culture, biofilms were observed using a confocal microscope at a 400× magnification.

It has been shown that certain fatty acid signals can act as agents to reduce, or negatively impact biofilm formation in several bacteria as demonstrated with 11-Me-C_12_:Δ^2^, 11-Me-C_12_:Δ^2,5^, and 12-Me-C_14_ [97,99,117]. It is therefore not surprising that *cis*-DA can also prevent biofilm formation, likely by keeping the cells in a constant metabolically active and/or dispersive growth state, as *cis*-DA also induces dispersion and increases the persister cells’ metabolic status. In a *S. aureus* MRSA strain, inhibition of bacterial growth was achieved in the presence of 2.94 mM of *cis*-DA, while as little as 734 μM *cis*-DA prevented biofilm formation [109]. Similarly, *cis*-DA prevented biofilm formation by single and dual-species biofilms of *E. coli* and *K. pneumoniae* in catheters [110], and in biofilms of *P. aeruginosa* PAO1 grown in flow cell reactors [19], when used at concentrations of 310 nM and 2.5 nM respectively. Continuous exposure of *P. aeruginosa* PAO1 expressing green fluorescent protein (GFP), in flow cell reactors to Electric Power Research Institute (EPRI) medium supplemented with 1 nM, 1 μM or 1 mM of *cis*-DA (Figure 1B–D, Table 2) under the conditions described by Davies and Marques [19], resulted in a significant reduction of maximum biofilm thickness (2-fold), average biofilm thickness (2.2-, 1.4- and 8-fold lower, respectively), roughness coefficient (1.5-fold lower, 6.4-fold lower and no-fold change, respectively), and total biomass (2.4-, 1.7- and 8.5-fold lower respectively), when compared to the absence of *cis*-DA (Figure 1, Table 2). Interestingly, *cis*-DA effectiveness does not increase with increasing concentration. Challenge of bacteria with 1 μM *cis*-DA (Figure 1C) actually resulted in higher biomass and increased microcolony average thickness than 1 nM *cis*-DA (Figure 1B), demonstrating that the effects of this fatty acid are due to its activity as a signaling molecule and not due to direct effects of the fatty acid as a detergent. However, increasing the *cis*-DA concentration to the mM range (Figure 1D), its effectiveness is once again present, indicating that at high concentrations *cis*-DA might have a detergent effect.

**Table 2:** COMSTAT analysis of *P. aeruginosa* PAO1 harboring the green fluorescence protein expressing vector pMRP9-1, grown in flow cell reactors in EPRI media, in the continuous presence of different concentrations of *cis*-DA. Results are the average of at least 4 replicates.

Biofilm Structure Quantification	Control	*cis*-DA 1 nM	*cis*-DA 1 μM	*cis*-DA 1 mM
	Average ± SD	Average ± SD	Average ± SD	Average ± SD
Total biomass (μm^3^/μm^2^)	20.66 ± 7.02	8.49 ± 2.38	12.29 ± 3.5	2.42 ± 0.86
Average thickness (μm)	26.46 ± 14.99	12.21 ± 3.73	19.37 ± 1.92	3.31 ± 1.19
Maximum thickness (μm)	146.13 ± 27.69	61.38 ± 13.22	50.80 ± 6.35	59.27 ± 15.98
Roughness coefficient (dimensionless, range: zero-infinity)	1.40 ± 0.38	0.90 ± 0.27	0.22 ± 0.05	1.86 ± 0.07

## 5. Conclusions

During the past 15 years, efforts to develop new and effective anti-biofilm chemotherapeutics has focused to a large degree on blocking the mechanism of signal transduction involved in cell-to-cell communication via quorum sensing, and has led to the development of several QS antagonists. The potential chemotherapeutic use of *cis*-DA differs significantly from existing anti-biofilm strategies, all of which exert a negative effect upon biofilm bacteria and are likely to be both limited in the spectrum of bacteria they act upon and likely to lead to some form of resistance. The biofilm dispersion response is undoubtedly necessary for the long-term survival of bacterial biofilm populations/communities. Thus, the development of resistance to autoinduction of biofilm dispersion is not likely to be an advantage to a bacterial population. This approach to infection management, therefore, presents a new direction in microbial control. One of the key advantages to the potential use of *cis*-DA in therapeutic contexts is the multiple desirable effects it induces in bacteria, inhibiting biofilm development, altering virulence, inducing the disaggregation of the biofilm by inducing a dispersion response, and reverting persister cells to a susceptible state. Studies with this signal have also shown that *cis*-DA has no bactericidal activity nor does it induce cytotoxic effects in fibroblasts. Instead, *ci*s-DA increases cell recovery on agar plates while reducing the number of persister cells formed in a population. Using *cis*-DA as an adjunctive to antimicrobial agents enhances killing of biofilm bacteria and persister cells, in some instances to the point of eradication. This effect is achieved by overcoming the mechanisms that lead to biofilm tolerance including: penetration failure, reduced metabolic rates and slow growth, biofilm-specific resistance (such as is mediated by the activation of efflux pumps), and the existence of a persister cell sub-population. Considering the urgent need to find new therapies to effectively treat chronic and unresolved infections, *cis*-DA provides a promising new potential chemotherapeutic to treat biofilm infections. Further research on this novel cell-to-cell signal is likely to elucidate the mechanism of signal transduction and reversion of persistence in cells challenged with *cis*-DA, as well as, reveal other molecules with similar activity and extend the list of microorganisms that use cell-to-cell signaling to induce biofilm dispersion.
